# Age-Related Neuroprotection by Dietary Restriction Requires OXR1-Mediated Retromer Function

**DOI:** 10.1093/geroni/igab046.2151

**Published:** 2021-12-17

**Authors:** Kenneth Wilson, Sudipta Bar, George Brownridge, Jennifer Beck, Rachel Brem, Hugo Bellen, Lisa Ellerby, Pankaj Kapahi

**Affiliations:** 1 Buck Institute for Research on Aging, Novato, California, United States; 2 Buck Institute for Research on Aging, Buck Institute for Research on Aging, California, United States; 3 University of California, Berkeley, Berkeley, California, United States; 4 Baylor College of Medicine, Houston, Texas, United States

## Abstract

Dietary restriction (DR) is the most robust method to delay aging and the onset of neurogenerative disorders across multiple species, though the mechanisms behind this phenomenon remain unknown. To elucidate how DR mediates lifespan extension, we analyzed natural genetic variants that associate with increased longevity under DR conditions in the Drosophila Genetic Reference Panel. We found that neuronal expression of the fly homolog of human Oxidation Resistance 1 (OXR1) is necessary for DR-mediated lifespan extension. Neuronal knockdown of OXR1 also accelerated visual decline but not physical decline, arguing for a specific role of OXR1 in neuronal signaling. Further, we find that overexpression of the TLDc domain from human OXR1 is sufficient for lifespan extension in a diet-dependent manner. Studies from the Accelerating Medicines Partnership - Alzheimer's Disease network show that patients with reduced OXR1 protein levels are more prone to Alzheimer's disease diagnosis, and we find that overexpression of human OXR1 is protective in animal and cell Alzheimer's models. In seeking the mechanism by which OXR1 protects against age-related neuronal decline, we discovered that it provides a necessary function in regulating the neuronal retromer complex, which is essential for the recycling of transmembrane receptors and for maintenance of autophagy. We further discovered that OXR1 deficiency can be rescued by genetic or pharmacological enhancement of retromer function, and that this enhancement extends lifespan and healthspan. Understanding how OXR1 operates could help uncover novel mechanisms to slow neurodegeneration including Alzheimer's disease.

